# Supplementary material for: Dental health assessed using panoramic radiograph and adverse events in chronic kidney disease stage 4–5 patients transitioning to dialysis and transplantation–A prospective cohort study
Source: PLoS One. 2021 Sep 30;16(9):e0258055. doi: 10.1371/journal.pone.0258055 (PMC8483312; doi:10.1371/journal.pone.0258055)
Supplement: S1 Table — (DOCX) [file pone.0258055.s001.docx]

S1 Table. Blood culture findings

| **Pathogen** | **Number of patients with positive cultures (%)** |
| --- | --- |
| *Escherichia coli* | 7 (32) |
| *Staphylococcus aureus* | 4 (18) |
| *Streptococcus dysgalactiae* | 3 (14) |
| *Staphylococcus epidermidis* | 2 (9) |
| *Streptococcus mitis* | 1 (5) |
| *Streptococcus constellatus* | 1 (5) |
| *Streptococcus lutetiensis* | 1 (5) |
| *Raoultella ornithinolytica* | 1 (5) |
| *Citrobacter braakii* | 1 (5) |
| *Klebsiella pneumoniae* | 1 (5) |
| *Stenotrophomonas maltophilia* | 1 (5) |
| *Sphingomonas koreensis* | 1 (5) |
| *Parvimonas micra* | 1 (5) |
| *Ruminococcus* | 1 (5) |
| *Bacteroides fragilis* | 1 (5) |
| *Clostridium perfringes* | 1 (5) |
