# Supplementary material for: Dental health assessed using panoramic radiograph and adverse events in chronic kidney disease stage 4–5 patients transitioning to dialysis and transplantation–A prospective cohort study
Source: PLoS One. 2021 Sep 30;16(9):e0258055. doi: 10.1371/journal.pone.0258055 (PMC8483312; doi:10.1371/journal.pone.0258055)
Supplement: Attachment — Submitted filename: Response to Reviewer.docx [file pone.0258055.s002.docx]

Editor Denis Bourgeois

PlosOne

September 14, 2021

**Re: #MS PONE-D-21-11023 Dental Health Assessed Using Panoramic Radiograph and Adverse Events in Chronic Kidney Disease Stage 4-5 Patients Transitioning to Dialysis and Transplantation –A Prospective Cohort Study.**

Dear Professor Bourgeois,

Thank you for your letter of September 14 concerning this manuscript. In our prospective observational study we investigated the association between radiographically assessed oral health at baseline and all-cause and cardiovascular mortality, major adverse cardiovascular events and symptomatic bacteremia during a 3-year follow-up in CKD stage 4-5 patients transitioning to dialysis and transplantation. Radiographically assessed dental health was independently associated with all-cause and cardiovascular mortality and MACEs but not with the incidence of bacteremia during follow-up.

We believe that that we have been able to respond to each of the comments of the reviewers, as outlined below, and are therefore grateful for the opportunity to resubmit the revised manuscript to PlosOne.

All authors have approved submission of the manuscript, and the manuscript has not been published and is not being considered for publication elsewhere. The authors do not have any conflicts of interest in connection with the submitted article.

Yours sincerely,

Adjunct professor Mikko Järvisalo, MD, PhD

Intensive care unit, Turku University Hospital

Building 18, TG3B

PO Box 52, 20521 Turku, Finland

Email: mikko.jarvisalo@tyks.fi

Phone: +358-2-3130049

Reviewer #1: Thank you for the changes made in a rigorous manner. Limitations of the study are well highlighted which is important. Conclusion provides recommendations for improving patient management

Reviewer #2: Thank you for this new submission which made important changes in line with our comments. Your document is much clearer and more precise (introduction - statistics - results).

Methods - study protocol: there were “no regular follow-up by a dentist in the study protocol”.

Specify that this is a bias in your discussion, especially on the data identified in the follow-up. Especially since you don't take new x-rays.

**We agree with the reviewer and have revised the Limitations section of the discussion accordingly to include this point. (Page 11, Paragraph 2, Lines 254-258)**

Methods - dental radiographs: Reading your paragraph, I understand that in the end only 171 patients were able to benefit from a PRD? So specify the final inclusion number (190? 171?).

**The correct number is 190. We have revised the text to clarify the number of patients with DPR and included in the analyses. (Page 5, Paragraph 2, Lines 99-104)**

Specify the statistical test used for the inter-intra observer variance.

**We have included this point to the Statistical methods section of the manuscript (Page 6, Paragraph 3, Lines 133-136)**

Discussion: Provide a hypothesis / justification / consequence to your new result regarding the outcome of bacteremia, and the weak relation to oral origin. This is major information.

**We have included the point that only a minor proportion of the bacteremias were of oral origin, which also makes it understandable that we did not observe an association between DPR findings and incident bacteremia. (Page 10, Paragraph 4, Lines 242-244)**

There is, for me, a difference between individual dental prophylaxis and antibiotic prophylaxis as you are only discussing. To review. Because the bacteremia has a clear influence depending on the availability of individual dental prophylaxis or not (many publications on this subject).

**We agree with the reviewer but unfortunately these data were not available for the current study. We have included this point to the revised manuscript. (Page 11, Paragraph 1, Lines 250-253)**

"No significant differences between patients with or without diabetes in any of the DPR measures": I think the rationale is rather that poor periodontal health is a risk factor for worsening diabetes, and therefore that regular information and monitoring are needed in these patients, and not the other way around as you assume (although diabetes is also a risk factor for periodontal disease).

**The reviewer is right. We have revised the text accordingly. (Page 10, Paragraph 3, Lines 233-236)**

**We would like to thank the reviewers for constructive criticism and comments and feel that the manuscript has improved by the changes made. Therefore, we hope that you will consider this revised manuscript acceptable for publication in PlosOne.**
